# Developing criteria for research translation decision-making in community settings: a systematic review and thematic analysis informed by the Knowledge to Action Framework and community input

**DOI:** 10.1186/s43058-022-00316-z

**Published:** 2022-07-16

**Authors:** Marilyn E. Wende, Sara Wilcox, Zoe Rhodes, Deborah Kinnard, Gabrielle Turner-McGrievy, Brooke W. McKeever, Andrew T. Kaczynski

**Affiliations:** 1 Department of Health Promotion, Education, and Behavior, Arnold School of Public Health, University of South Carolina, Discovery I, Suite 565, 915 Greene St., Columbia, SC 29201 USA; 2 Prevention Research Center, Arnold School of Public Health, University of South Carolina, 921 Assembly Street, Columbia, SC 29208 USA; 3 Department of Exercise Science, Arnold School of Public Health, University of South Carolina, 921 Assembly Street, Columbia, SC 29208 USA; 4 School of Journalism and Mass Communications, College of Information and Communications, University of South Carolina, 800 Sumter Street, Columbia, SC 29208 USA

**Keywords:** Research translation, Translation, Criteria, Community settings, Theoretical frameworks

## Abstract

**Background:**

There is a pressing need to translate empirically supported interventions, products, and policies into practice to prevent and control prevalent chronic diseases. According to the Knowledge to Action (K2A) Framework, only those interventions deemed “ready” for translation are likely to be disseminated, adopted, implemented, and ultimately institutionalized. Yet, this pivotal step has not received adequate study. The purpose of this paper was to create a list of criteria that can be used by researchers, in collaboration with community partners, to help evaluate intervention readiness for translation into community and/or organizational settings.

**Methods:**

The identification and selection of criteria involved reviewing the K2A Framework questions from the “decision to translate” stage, conducting a systematic review to identify characteristics important for research translation in community settings, using thematic analysis to select unique research translation decision criteria, and incorporating researcher and community advisory board feedback.

**Results:**

The review identified 46 published articles that described potential criteria to decide if an intervention appears ready for translation into community settings. In total, 17 unique research translation decision criteria were identified. Of the 8 themes from the K2A Framework that were used to inform the thematic analysis, all 8 were included in the final criteria list after research supported their importance for research translation decision-making. Overall, the criteria identified through our review highlighted the importance of an intervention’s public health, cultural, and community relevance. Not only are intervention characteristics (e.g., evidence base, comparative effectiveness, acceptability, adaptability, sustainability, cost) necessary to consider when contemplating introducing an intervention to the “real world,” it is also important to consider characteristics of the target setting and/or population (e.g., presence of supporting structure, support or buy-in, changing sociopolitical landscape).

**Conclusions:**

Our research translation decision criteria provide a holistic list for identifying important barriers and facilitators for research translation that should be considered before introducing an empirically supported intervention into community settings. These criteria can be used for research translation decision-making on the individual and organizational level to ensure resources are not wasted on interventions that cannot be effectively translated in community settings to yield desired outcomes.

Contributions to the literature

1. This systematic review, informed by the Knowledge to Action Framework, created a list of criteria to assist researchers, practitioners, and community partners in evaluating intervention readiness for translation into community and/or organizational settings.
2. The resultant criteria can be used to ensure resources are not wasted on interventions that cannot be effectively translated to yield desired outcomes and provide more consistency in the research translation process.
3. This study expands on burgeoning research focused on the decision to translate interventions since there is a need for more research on this pivotal juncture between academic knowledge and community settings.

## Introduction

The World Health Organization (WHO) estimates that chronic diseases account for 71% of mortality globally, amounting to 41 million deaths each year [1, 2]. Addressing increasing rates of chronic disease is important to improving health, healthcare spending, and quality of life [3–5]. To optimize the use of public health funding, public health and community-oriented practitioners should focus on evidence-based interventions, products, and policies that show promise for implementation in community settings [6]. There is a pressing need to translate empirically supported interventions, products, and policies into practice to prevent and control prevalent chronic diseases.

The Knowledge to Action (K2A) Framework is useful for understanding and informing the process of how research is translated into practice and ultimately institutionalized in public health and community settings [7]. The Centers for Disease Control and Prevention’s (CDC) Work Group on Translation, comprised of various divisions within the National Center for Chronic Disease Prevention and Health Promotion, created the K2A Framework [7]. The K2A Framework describes three phases necessary to move knowledge into sustainable action, including the research phase (e.g., efficacy and effectiveness research), the translation phase (e.g., decision to translate knowledge into products, decision to adopt), and the institutionalization phase (e.g., establishing the intervention activities, creating norms within communities) [7, 8]. Moreover, K2A describes the importance of supporting structures within each phase as well as evaluation to move discovery into action [8]. The framework was designed to be relevant regardless of the disease, condition, or risk factor being addressed. K2A also applies to many types of evidence-based programs, policies, interventions, guidelines, tool kits, strategies, and/or messages (hereafter referred to as “interventions”) and works best if research and practice communities are in collaboration [7, 8].

The K2A Framework identifies the “decision to translate” as a pivotal transition step from the research phase to the translation phase. In this step, those responsible for developing or testing the intervention (often the researchers) and those in organizations likely to use them decide whether there is adequate evidence to create an actionable product or to propel an evidence-based intervention into widespread use [8]. Only those interventions deemed “ready” for translation are likely to be disseminated, adopted, implemented, and ultimately institutionalized. Yet, this pivotal step has not received adequate study [9]. Although the K2A framework does have some shortcomings, including that it specifically focuses on evidence-based programs, practices, and policies, and somewhat ignores research products (e.g., tools, indices, technologies) and that it largely targets public health professionals (ignoring the potential use for practitioners or community stakeholders), it is a landmark tool for guiding research translation [8].

In the CDC’s 2018 Request for Applications for Health Promotion and Disease Prevention Research Centers, applicants were required to describe how they would undertake three activities related to translation: (1) propose a prevention research and translation agenda, (2) engage translation partners to increase the translation of research findings into public health practice, and (3) conduct activities to support the translation of center products. In response to the second and third required activities, the University of South Carolina Prevention Research Center proposed to establish criteria to determine if a product or program resulting from our Center’s research is ready for translation and to solicit feedback from community stakeholders on the criteria. These criteria can then be used to prioritize interventions for translation, determine what adaptations may be necessary for translation into a specific setting, and determine whether additional training or resources are needed for the intended audience.

This paper describes the process used to establish criteria for the decision to translate and highlights considerations that can be used to evaluate or compare empirically supported interventions and their readiness for translation. Much has been published in the field of implementation science about factors that influence the adoption, implementation, and dissemination of evidence-based interventions [10–19]. Some of these factors likely apply to the decision to translate interventions, while others may not. Furthermore, unlike models that guide the study of adoption, implementation, and dissemination, comprehensive frameworks or models do not appear to exist to help researchers and organizational partners decide which interventions are most suitable for translation [13]. Nonetheless, establishing criteria for the decision to translate can be informed by several areas of investigation, including “designing for dissemination,” [20] consideration of specific intervention characteristics that facilitate research translation and dissemination [21–24], and review of factors important for implementation [15–17, 25, 26]. Such criteria are especially needed in community-based research, where researchers, public health-related agencies, and organizations collaborate. The voices of community partners are critical in this process, as they can enhance the quality and relevance of translated research [27].

The purpose of this paper was to create a holistic list of criteria that can be used by researchers, in collaboration with community partners, to help evaluate intervention readiness for translation into community and/or organizational settings. Specifically, research objectives include (1) systematically review existing literature focused on factors influencing translation of empirically supported interventions into community settings and (2) develop a list of factors that have influenced community translation of empirically supported interventions in past research that can be used as criteria for the decision to translate. Since the K2A framework is a well-known planning tool that uniquely highlights the decision to translate and other key aspects of the research translation phase [7], it was used to inform this systematic review. Specifically, the K2A Framework was a starting point in this process and was augmented by a systematic literature review combined with consideration of researcher and community partner input.

## Methods

The identification and selection of criteria began with a review of K2A questions from the “decision to translate” stage and then involved conducting a literature review to identify characteristics important for research translation in community settings, using thematic analysis to select unique research translation decision criteria, and soliciting and incorporating researcher and community advisory board feedback.

### Knowledge to Action (K2A) Framework

The “decision to translate” stage of the K2A Framework includes 8 planning questions to help decide whether an intervention is ready to move forward for translation (e.g., Is this intervention needed? Is there broad support or buy-in to translate the intervention into practice?) [8]. These questions were converted to items on our coding manual and were used as a starting point for developing criteria for the decision to translate research.

### Literature review search strategy

Our next step was to conduct a systematic literature review to understand criteria or factors presented in past research that were relevant to research translation. The review included academic literature identifying criteria for deciding which empirically supported interventions could be effectively translated into practice as well as articles that more generally identify factors relevant to the translation process. PubMed and Google Scholar were searched for peer-reviewed articles published between January 2000 and August 2020. The primary goal of this review was to identify the most prominent literature on research translation and related decisional criteria, so we did not undertake the time-consuming task of integrating a comprehensive list of academic databases. Although some published research identifying factors that impact research translation may not have appeared in our searches, it is unlikely that critical criteria were missed that were not captured by these two databases. PubMed contains more than 32 million citations for biomedical literature from MEDLINE, life science journals, and online books, and Google Scholar contains 389 million records and is currently the most comprehensive academic search engine [28, 29].

A broad initial literature search was performed to identify common terms related to our review and to develop the final search strategy. In turn, we developed and used the following search terms: ‘translation’ OR ‘knowledge translation’ OR ‘integrated knowledge translation’ OR ‘research translation’ OR ‘research to translation’ OR ‘translation decision’ AND (‘criteria’ OR ‘decision’ OR ‘designing for dissemination’ OR ‘community setting’) under the category “Title/Abstract” between January 2000 and August 2020. We chose to only include publications after the year 2000 to ensure our findings on community interventions were relevant and to reflect important developments in the field of implementation science (e.g., creation of frameworks for community implementation) [7, 30, 31]. In the search terms, we also included names of prominent authors that have published on the topic of research translation to identify additional relevant papers.

### Process for literature review study selection

Eligibility criteria included (1) research with human subjects, (2) research published in English, (3) research published between 2000 and 2020, (4) peer-reviewed articles or academic literature, and (5) research conducted in community settings. Specifically, community-based research was the focus of this review due to its relevance to our research team, and because the differences in the application and scope of research translation between community versus clinical settings can be substantial. Community settings were defined as “settings for which the primary purpose is not medical care, for example, geographic communities, schools, churches, homeless shelters, worksites, libraries” [32]. Research in community settings can include studies conducted over the phone, online, or in-person that engage groups of people or organizations that are defined by a function, geography, shared interests, or specific characteristics [33].

Titles and abstracts of articles identified through the search strategy were imported into Zotero, and duplicates were removed. Articles were evaluated for eligibility based on the criteria previously stated (i.e., community setting, human subjects, including criteria that could inform the decision to translate research) by a first reviewer (MW). An additional reviewer (SW), with substantial expertise in the field of research translation, provided input throughout the process towards improving the search terms and identifying relevant articles. At the critical full-text review stage, remaining articles were independently screened for eligibility by the first reviewer (MW), while a second reviewer (ZR) screened all articles using the same eligibility and inclusion criteria. One reviewer (MW) compiled data from each article in a Microsoft Excel database, including titles/authors, publication year, objectives, translational products, criteria/factors identified as important for community translation, and literature gaps addressed. Secondary reviewers (SW, ZR) checked data entry for accuracy and completeness. Discussions were held among all reviewers to resolve any remaining conflicts on article inclusion.

### Criteria development and selection

The criteria identified from the literature review, including criteria from the K2A Framework that were also noted in selected articles, were combined to form a full list of potential criteria. One researcher (MW) reviewed the full-text articles identified in the literature review for the presence of the constructs related to the decision for research translation of interventions. The researcher used a deductive and inductive thematic analysis approach, guided by constructs of the K2A framework (i.e., codes related to importance of evidence base, relation to a high-priority public health issue, alignment with constituent needs, comparability to other interventions, support or buy-in for translation, presence of supporting structures, economic considerations, adaptability to contextual changes) [34–36]. Emergent themes included any factors that past literature highlighted as important for research translation or dissemination or factors that specifically impacted research translation or dissemination for a certain intervention in practice. Each article was independently reviewed by one researcher (MW), who met periodically throughout the entire article review process to discuss findings with a second expert (SW) and adapt the review process when necessary. In these discussions, the researchers decided that themes that identify intervention *and* population-level barriers or facilitators to research translation should be included in the criteria list. As an example, some studies reported results from the evaluation of interventions and noted decisions that were made about how to move a study from a controlled pilot to wide-scale dissemination. Other articles described the contextual factors that were advantageous for translating research into community practice.

These resultant criteria were subsequently reviewed by three researchers and members of the University of South Carolina Prevention Research Center Community Advisory Board to improve the language and ensure all criteria were relevant and no criteria were overlooked. All three researchers had familiarity or expertise in research translation and/or community-based participatory research. Thirteen Community Advisory Board members were from diverse sectors, including faith-based organizations, public health, economic and community development, physical activity and healthy eating coalitions, and community-based research initiatives. Community Advisory Board members were led through a process where they generated factors that are most important in their decision to use evidence-based programs, reviewed the criteria developed by the Center, and provided input as to whether any criteria were missing, should be removed, or should be revised. Since these criteria were being developed for use by our university’s Prevention Research Center, incorporating community and research input from nationally representative locations was outside the scope of this study.

### Data synthesis

Upon receiving community and expert input on the research translation decision criteria identified through our systematic review, we identified the frequency of articles mentioning each and ordered our criteria list according to the most cited (i.e., each item’s usefulness in past research). We also identified underlying frameworks used in each article to guide translation/implementation research, since this may have influenced the respective authors’ focus when outlining possible research translation criteria or factors. Lastly, we identified the frequency in which articles mentioned research translation criteria that were part of the K2A framework (the framework orienting this research).

## Results

### Study selection — objective 1

**Fig. 1:**
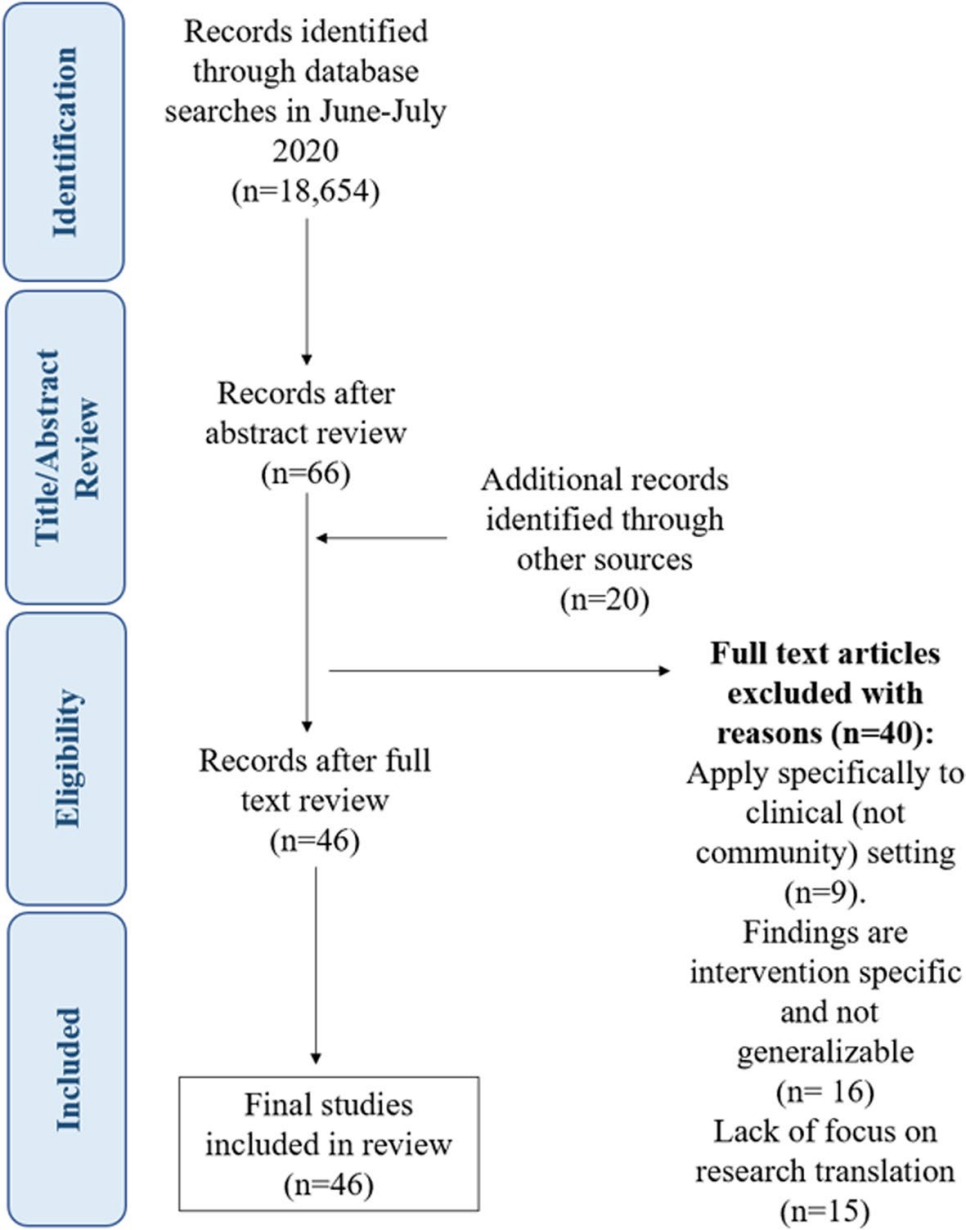
PRISMA diagram of included and excluded studies, with reasons for inclusion

As shown in Fig. 1, the search terms returned 18,654 results, and a total of 66 relevant articles were included after an abstract review. After reviewing the reference lists in the selected articles and incorporating papers from other sources, 20 additional articles were identified as relevant to this review. Two reviewers (MW and ZR) screened full-text articles using the same eligibility and inclusion criteria. Their agreement rate was 88%. After a full-text review of the 86 articles identified, 40 articles were removed because they did not contain findings specific to community settings and/or did not focus on research translation. A final list of 46 articles was used to develop the research translation decision criteria for this article (Fig. 1). Information was compiled for each article identified in our search about the interventions that were being translated and the criteria that might be useful for research translation decisions (Table 1).

**Table 1:** Research translation decision criteria described by the papers included in the review (*N* = 46)

Author(s), (year), and reference	Objectives	Translational item(s)	Relevant study findings or commentary	List of community-based translational decision criteria used (see Table 2)
Glasgow (2003) [37]	Authors use the RE-AIM framework to illustrate challenges inherent in translation of diabetes care interventions.	Evidence-based findings for diabetes care	Methods/factors that can accelerate the translation of research to practice: (1) enhance and measure the reach of interventions, especially toward poor, underserved, and minority populations; (2) develop programs that can be widely adopted by diverse settings; (3) produce replicable effects and enhance quality of life, in addition to short-term behavioral or biological outcomes; (4) be consistently implemented by different staff members having moderate levels of training; and (5) produce maintenance at both individual and setting levels and at reasonable cost.	#1 #9 #10 #17
Glasgow et al. (2004) [22]	This article highlights reasons why most behavioral and health promotion studies have not been translated into practice, focusing on study design characteristics as a central contributing barrier.	Health promotion studies	Criteria to improve research translation include (1) feasibility and costs of interventions, (2) assessments of what works across various targeted groups, under assorted conditions/diverse settings (i.e., context-specific efficacy research), (3) adequate time for translation and sufficient necessary resources, and (4) absence of competing demands. Also, changes will be required on the part of researchers, funding agencies, and review/editorial boards.	#1 #2 #6 #7 #9
Dzewaltowski et al. (2004) [38]	Authors describe individual- and setting-level factors important for translation that moderate the impact of interventions and often are not reported in the literature.	Health promotion research on physical activity engagement	Individual participant-level indicators that impact research translation include reach and efficacy. Setting-level indicators include adoption and implementation. Maintenance is assessed at both an individual and setting level of impact. In addition, consideration of internal and external validity in the planning, design, and evaluation of health behavior promotion interventions.	#1
Glasgow et al. (2004) [39]	Authors discuss issues in, barriers to, and lessons learned regarding the dissemination of interventions. Specifically, they summarize previous reviews, exemplary studies, and theories on the diffusion/dissemination of cancer screening interventions.	Research on efficacious cancer screening programs	Six lessons learned: address the involvement of key stakeholders, factors influencing diffusion, the need for different types of efficacy and effectiveness studies with greater attention to external validity, replication, use of theoretical and evaluation models, and importance of policy infrastructure.	#1 #5 #6
Klesges et al. (2005) [40]	This article describes the RE-AIM framework and how it can be used to plan and design studies with features that can strengthen the potential translation of interventions.	Behavioral change interventions	Considerations for research translation: (1) studies of external validity in less controlled and optimal contexts are important (and often overlooked since internal validity is easier to achieve); (2) adoption rate (including ineligibility of certain settings/populations, representation); (3) potential for maintenance of intervention by research staff or community; (4) consideration of “how” and “why” and intervention might be adopted into practice.	#1 #5 #8 #14
Green and Glasgow (2006) [41]	This article suggests criteria to evaluate intervention external validity and potential for generalization and recommends procedures to adapt interventions and integrate them with evidence on population and setting characteristics, theory, and experience into locally appropriate programs.	Practice-based evidence	Considerations for research translation: (1) program adaption and evolution/maintenance; (2) attention to issues of practice-based, real-time, ordinary settings and related research (instead of just basing research translation on the strongest controlled evaluations); (3) external validity should be considered to the same degree as internal validity.	#1 #4 #8 #10
Glasgow and Emmons (2007) [42]	This review summarizes factors that have interfered with translation of research to practice and what public health researchers can do to hasten such transfer.	Evidence-based, efficacious interventions	Intervention characteristics that impact research translation: (1) high cost, intensive time demands; (2) high level of staff expertise required; (3) difficult to learn/understand; (4) not packaged or “manualized”; (5) not developed considering user needs, not designed to be self-sustaining; and (6) highly specific to a particular setting, not modularized or customizable. Target setting characteristics that impact research translation: (1) competing demands, program imposed from outside; (2) financial/organizational instability, specific needs of clients/setting; (3) limited resources, time, and organizational support; (4) prevailing practices work against innovation; (5) perverse incentives/regulations; (6) challenges implementing interventions with quality.	#3 #5 #6 #8 #9 #10 #14 #15 #16
Prohaska and Peters (2007) [43]	This paper discusses problems that might be faced when translating basic research findings into public health practice for cognitive impairment and/or dementia in older adults and addresses how some of these problems might be overcome.	Research on physical activity among the elderly	The impact of research translation and dissemination is evaluated in terms of (1) the depth and range of settings in which the research innovation and public health message are adopted, (2) the degree to which the programs and innovations are implemented as intended, and (3) the degree to which the research is maintained and institutionalized in these settings.	#10 #14 #16
Baker et al. (2008) [21]	The purpose of this article was to describe the identification and evaluation of research- and practice-based evidence criteria.	Public health evidence	Research- and practice-based evidence criteria: (1) context (political, social, or economic), (2) changeability of intervention, (3) community readiness, ability to evaluate, (4) resources, (5) time, and cost constraints, (6) intervention is succinctly described, (7) replicability of procedures, (8) sustainability, (9) adaptability, (10) fidelity, (11) feasibility, (12) reach, (13) intervention acceptability across multiple populations, (14) tools and protocols are available for public use, level of support needed (i.e., technical assistance), (15) simplicity, (16) multiple structured activities, (17) practicability without funding, (18) leadership, (19) organizational commitment, (20) community engagement, (21) partnership factors (size, history, communication/trust, compatibility of organizational cultures)	#3 #5 #6 #9 #10 #13 #15
Flaspohler et al. (2008) [44]	This article operationalizes capacity and distinguishes among types and levels of capacity as they relate to dissemination and implementation.	Theory and research findings	Organization-specific capacity factors: (1) fit with goals, values, norms, practices, and organizational and program needs; (2) ability to adapt to suit needs and select appropriate innovations; (3) selection of staff to implement innovation, strong administrative support, formal commitment (i.e., to provide necessary resources); (4) employee skill, incentives, limited obstacles, support for staff in implementation (training/support); (5) assistance for sustainability of innovation; and (6) staff agreement on program values. In addition, strategic placement of supporters in the organization, well-connected local champion, credibility of program within community, potential for program sustainability, and technical capacities/assistance are important.	#3 #5 #6 #10 #14
Guerra and Knox (2008) [45]	The article examines the impact of cultural characteristics on the translation of innovations into practice at the community level, relying on an interactive systems framework.	Evidence-based family-school interventions/programs	For research translation, conditions for utilization of support must be consistent with the cultural framework of the agency and the community. Partnerships should be understood as “expert” advice from a partner who is trustworthy, competent, and knowledgeable about local concerns and cultural norms, as part of an ongoing relationship that would endure over time.	#11 #12
Livet et al. (2008) [46]	This article examines types of organizational characteristics that are related to the successful use of programming processes (i.e., planning, implementation, valuation, and sustainability) that are part of comprehensive programming frameworks.	Comprehensive programming frameworks (e.g., Communities that Care-CtC)	Except for sustainability, process-specific organizational capacities were more highly correlated with programming process use than overall organizational functioning variables. The strongest and most consistent factor related to the use of all planning steps was the presence of a planning process champion. The amount of technical assistance and training and the presence of an implementation process champion were the only variables related to the extent of use of implementation guidelines. Insufficient financial resources were the only factor that strongly correlated with greater implementation of evaluation guidelines.	#5 #6 #9 #14
Wandersman et al. (2008) [47]	This article presents the Interactive Systems Framework for Dissemination and Implementation that uses aspects of research to practice models and of community-centered models.	Evidence-based practices in medicine, public health, and psychotherapy treatment	Overarching criteria that impact evidence translation: (1) funding, (2) macro policy, (3) climate, and (4) existing research and theory. Within that, there is the “system of implementing prevention” (or the prevention delivery system) that is impacted by general capacity and innovation-specific capacity use. Another system within is “supporting the work” (or prevention support system) impacted by general capacity building, and innovation-specific capacity building. The last system within is “distilling the information” (or prevention synthesis and translation system) which is impacted by synthesis and translation.	#1 #4 #5 #6 #7 #10
Scharff and Mathews (2008) [48]	This article describes the importance of community engagement throughout the translation and dissemination process, strategies for increasing it, and the steps that need to occur so that community participation is recognized and utilized.	Scientific discoveries	The use of community-based participatory research requires that researchers be culturally competent, that is, they demonstrate respect for individual and cultural differences and implement trust promoting methods of inquiry. Three components of cultural competency: knowledge, attitude, and skill. Being knowledgeable about communities, understanding world views and cultural context of communities. Researchers recognize and address their own prejudices/stereotypes. Use of a set of skills, including listening and other communication skills that can help create and sustain relationships.	#11 #12
Durlak and DuPre (2008) [14]	This review assesses the impact of implementation on program outcomes and identifies factors affecting the implementation process.	Health promoting interventions	Organizational factors that impact implementation: (1) need for community structure (e.g., a coalition) or an existing community-based agency (e.g., health clinic, community service center) and (2) training and technical assistance that is provided by outside parties. Ecological context factors that impact implementation: (1) innovation characteristics, (2) provider characteristics, (3) community factors, (4) the prevention research system, (5) politics/policy, and (6) funding.	#6 #10
Mendel et al. (2008) [33]	This paper describes the research questions and challenges that have arisen in the efforts to address health issues and the progressive succession of approaches taken to study organizational contexts in healthcare and community settings.	Evidence-based health interventions within community settings	Setting-level factors impacting research translation: (1) macro system environment, (2) legal/policy environment, (3) resource/economic environment, (4) cultural/normative environment, and (5) organizational networks and linkages. Important stakeholders: professional networks, communities of practice, social support networks, populations in need or at risk, care delivery organizations, interest groups, regulatory agencies, insurers, and purchasers. These all play a role in the context of diffusion (of the intervention) and should be evaluated before translation with a capacity/needs assessment. Once evaluated, adoption, implementation, and sustainment will occur to yield intervention outcomes.	#5 #6 #9 #11
Arrington et al. (2008) [49]	The purpose of this article is to report on the process and results of a year-long project designed to build a Missouri action plan for improving public health practice through increased research translation and dissemination.	Public health knowledge	Themes identified to improve research translation: (1) provide education and training; (2) enhance capacity; (3) change incentives and accountability; (4) shift funding toward community needs; (5) support practice-based research; (6) engage and collaborate with the community; (7) share knowledge; (8) engage influential people; and (9) sustain momentum; action plans were drafted to address priorities in each cluster.	#1 #5 #6
Saul et al. (2008) [50]	This article illustrates ideas for bridging science and practice generated during the Division of Violence Prevention’s dissemination/implementation planning process.	Research on youth violence prevention	To bridge science and practice, funders can promote the expectation that capacity-building efforts should be held to an evidence-based standard. Training, technical assistance, and coaching should be based on knowledge on the effectiveness of delivering such support, and those who provide it should be required to evaluate their effect on changes in practice. Resources should be made available for strong syntheses and translation of evidence-based approaches.	#6
Damschroder et al. (2009) [51]	Authors describe the CFIR that offers an overarching typology to promote implementation theory development and verification about what works, where, and why across multiple contexts.	Empirically supported interventions	Intervention characteristics that promote implementation: (1) intervention source (i.e., perception of key stakeholders on whether intervention is internally or externally developed), (2) evidence strength and quality, (3) relative advantage, (4) adaptability, (5) trialability, (6) complexity, (7) design quality and packaging, (8) and cost. Outer setting factors impacting implementation: (1) patient needs/resources, (2) cosmopolitanism, (3) peer pressure, and (4) external policies/incentives. Inner setting factors: (1) structural characteristics, (2) networks and communications, (3) culture, and (4) implementation climate. Individual characteristics: (1) knowledge/beliefs about the intervention, (2) self efficacy, (3) individual stage of change, (4) individual identification with organization, and (5) other attributes (e.g., intellect, motivation, values, capacity, learning style recognized by implementors).	#1 #3 #4 #5 #6 #7 #9 #10 #11
Prochaska et al. (2009) [52]	This article describes the process for developing the curriculum and application of Rogers’s Diffusion of Innovations theory and Glasgow and colleagues’ RE-AIM framework in guiding dissemination and evaluation of its adoption and implementation in psychiatry residency and graduate psychiatric nursing programs.	Evidence-based curriculum for tobacco cessation	Intervention barriers to adoption and implementation: (1) high cost, (2) intensive time demands, (3) high level of staff expertise required, (4) difficult to learn/understand, (5) not packaged or “manualized”, (6) not developed considering user needs, (7) not self-sustaining, (8) highly setting specific, and (9) not modularized/customizable. Adoption setting barriers to adoption and implementation: (1) competing demands, (2) program imposed from outside, (3) unstable finance or organizations, (4) clients and setting have specific needs, (5) limited resources/time, (6) limited organizational support, (7) prevailing practices that work against innovation, (8) incentives/regulations that oppose change, (9) characteristics of the research design, (10) not relevant or representative sample of patients, settings, or clinicians, (11) failure to evaluate cost/maintenance/sustainability and assess implementation.	#3 #6 #8 #9 #10 #14 #16 #17
Green et al. (2009) [53]	Authors review concepts that have guided or misguided public health in their attempts to bridge science and practice through dissemination and implementation.	Public health and medical knowledge	Five broad principles to bridge science to practice gap: (1) Needs of patients and populations should dictate the health research agenda; (2) Research agenda should address contextual and implementation issues including the development of implementation and accountability systems; (3) Research agenda should dictate the research methodologies rather than methodologies dictating the research agenda; (4) Researchers/practitioners/other users should collaborate to define the research agenda, allocate resources, and implement the findings; (5) Level of funding for dissemination and implementation research should be proportionate to the magnitude of the task.	#3 #9
Van Olphen et al. (2009) [54]	This evaluation used community-based participatory research guidelines to evaluate the participatory approach of the Community Outreach and Translation Core of the Bay Area Breast Cancer and the Environment Research Center in translating scientific findings.	Community-based participatory research	Identified approaches for translating scientific findings: (1) alignment of the project with principles of participatory research; (2) project structure as facilitator and barrier; (3) lack of explicit agreements regarding stakeholder roles; (4) nature and stage of research; (5) community involvement, (6) stakeholder skills, priorities, and needs tied to level of participation; (7) communication challenges the participatory process; and (8) lack of trust hinders participation.	#6 #5 #11
Ritzwoller et al. (2009) [55]	This paper introduces the Smoking Less, Living More study that was used as a prototype for their cost assessment methods, presents a five-step cost assessment guide designed for evaluation of the behavioral interventions, and reports results pertaining to the Smoking Less, Living More study and sensitivity analyses.	Behavioral interventions	Intervention costs must be distinguished from research, development, and recruitment costs. The inclusion of sensitivity analyses is recommended to understand the implications of implementation of the intervention into different settings using different intervention resources. Cost analysis can provide researchers and policymakers with valuable information regarding the feasibility of a proposed intervention.	#7 #9 #10
Sousa and Rojjanasrirat (2011) [56]	This paper reviews recommendations of cross-cultural validation of instruments/scales and proposes a guideline for the translation, adaptation, and validation of instruments/scales for cross-cultural health care research.	Instruments or scales for use in cross-cultural health care research	Research instruments must be properly translated to fit the community needs and maintain their same meaning. The instrument must show reliability and validity in cross-cultural research. Afterwards, pilot testing of the new instrument must be completed before translation, in general and in the target population.	#1 #3 #17
Meyers et al. (2012) [57]	The goal was to summarize research support that exists for the different steps in the quality implementation framework and offer suggestions for future research efforts. The practical goal was to outline the practical implications of our findings in terms of improving future implementation efforts in the world of practice.	Evidence-based research	Initial considerations regarding the host setting, assessment strategies: (1) needs and resources assessment, (2) fit assessment, and (3) capacity/readiness assessment. Decisions about adaptation: possibility for adaptation. Capacity-building strategies: (1) buy-in from critical stakeholders, (2) supportive community/organizational climate, (3) general/organizational capacity, (4) staff recruitment/maintenance, and (5) effective pre-innovation staff training. Creating a structure for implementation, structural features: implementation teams and plan. Ongoing structure once implementation begins: (1) support strategies, (2) technical assistance/coaching/supervision, (3) process evaluation, (4) supportive feedback mechanism, and (5) learning from experience.	#5 #6 #10
Cilenti et al. (2012) [58]	This article describes factors that contribute to successful translation of science into evidence-based practices and their implementation in public health practice agencies, based on a review of the literature and evidence from case studies.	Public health science	The CFIR was most applicable to case studies in this article. It organizes the constructs into 5 domains: (1) intervention characteristics (i.e., perceived source, evidence strength and quality), (2) outer setting (i.e., community needs/resources, (3) external policies/incentives, (4) inner setting (i.e., perceived need for change, available resources), and (5) characteristics of individuals (i.e., knowledge/beliefs about the intervention), implementation process (i.e., quality of planning/engaging staff). Commonly cited barriers to implementing evidence-based practices: (1) lack of time, (2) inadequate funding, and (3) absence of cultural and managerial support (including incentives).	#1 #3 #5 #6 #9 #12
Brownson et al. (2013) [20]	This paper describes the practice of designing for dissemination among researchers in the USA with the intent of identifying gaps and areas for improvement.	Public, environmental, and occupational health research	Factors to consider when designing for dissemination: (1) formal training/access to someone with formal training in communication, (2) dedicated person/team for dissemination in unit/organization, (3) unit/department has formal communication/dissemination strategy, (4) use of framework/theory to plan dissemination activities, and (5) frequent summaries for non-research audiences, stakeholders involved.	#6
Glasgow (2013) [59]	This paper provides examples of pragmatic methods, measures, and models and how they have been applied.	Scientific research	PRECIS model factors influencing research translation: (1) flexibility of the comparison intervention, (2) practitioner expertise, (3) flexibility of the experimental intervention, (4) eligibility criteria, (5) primary analysis, (6) practitioner adherence, (7) participant compliance, (8) outcomes, and (9) follow-up intensity. Recommended characteristics of measures/metrics: (1) reliability, (2) validity, (3) sensitivity to change, (4) feasibility, (5) importance to practitioners, (6) public health relevance, (7) actionable, user-friendly, (8) broad applicability, (9) cost, (10) ability to enhance patient engagement, and (11) causes no harm. Evidence Integration Triangle model factors influencing research translation: (1) internal and external validity and key components of the intervention/program/policy, (2) participatory implementation process (i.e., stakeholder engagement), and (3) practical progress measures (actionable, longitudinal measures). Multi-level context is important: intrapersonal, biological, interpersonal, organizational, policy, community, economic, social, and environmental factors.	#1 #2 #5 #10
Phillips et al. (2014) [60]	The article presents a research agenda to accelerate the dissemination/implementation of empirically supported physical activity interventions into care.	Empirically supported physical activity interventions into care for cancer survivors	Use of RE-AIM framework, which highlights internal and external validity. Intervention-specific barriers: (1) intense, high cost, on-site interventions in high-resource settings; (2) high level of expertise required; (3) inflexible programs; (4) do not meet the needs of survivors; (5) not “packaged” or manualized; and (6) need for participants to travel to participate. Setting-specific barriers: (1) lack of time/resources; (2) competing demands; (3) physical space restrictions; (4) limited staff with expertise; (5) limited organizational support; and (6) specific needs/situations limit ability to implement. Research design-specific barriers: (1) exclude those with other chronic conditions; (2) nonrepresentative, homogenous samples; (3) failure to evaluate cost/implementation; (4) do not involve stakeholders.	#1 #3 #6 #7 #8 #16
Cohen et al. (2015) [61]	This article presents the case study of “1-2-3 Pap,” a health communication intervention to improve human papillomavirus vaccination uptake and Pap testing outcomes in Eastern Kentucky, and explores strategies used to disseminate this intervention to other populations.	Communication intervention to improve HPV vaccine uptake	Considerations include (1) developing strategies for reaching other potential audiences, (2) identifying intervention message adaptations that might be needed, and (3) determining the most appropriate means or channels by which to reach these potential future audiences.	#10
Neta et al. (2015) [62]	This article presents and discusses implications of their framework to highlight areas that are underreported but would substantially enhance the value of research for end-users with the end goal of improving population health, and to compare concepts in existing reporting guidelines to our framework.	Public health research	Applied research is almost always multi-level and crosses social-ecological levels. This includes historical context, policy climate, and incentives, as well as organizational settings and persons delivering and receiving interventions, and is often lacking or missing important elements. The issue of “fit” or alignment (or lack of fit) between an intervention program or policy and its context is one of the key elements of the framework.	#3 #6
Ross et al. (2016) [25]	This review provides an update and re-analysis of a systematic review of the e-health implementation literature culminating in a set of accessible and usable recommendations for anyone involved or interested in the implementation of e-health.	E-health interventions in healthcare settings	Factors influencing implementation for e-health: (1) complexity, (2) adaptability, (3) compatibility with existing systems and work practices, (4) cost, (5) key stakeholders and implementation champions included as early in the implementation process, (6) sufficient financial and legislative support, (7) standards for technology (which address inter-operability, security, and privacy may improve acceptability/implementation), (8) organizations are in a state of readiness, (9) training and education, (10) ongoing monitoring, evaluation and adaptation of systems, (11) benefits realized, and (12) ongoing identification of barriers to effective use, along with strategies to overcome these barriers.	#4 #5 #6 #7 #8 #9 #15
Brownson et al. (2018) [63]	This article describes (1) lessons related to dissemination from related disciplines (e.g., communication, agriculture, social marketing, political science), (2) current practices among researchers, (3) key audience characteristics, (4) available tools for dissemination, and (5) measures of impact.	Public health knowledge	While public health practitioners value evidence-based approaches and dissemination, the heterogeneity of the workforce presents challenges. Studies among state public health practitioners have shown that only 46% use journals in their day-to-day work and use is lower (33%) at the local level. Lack of access is a major barrier to journal use. Other barriers to the use of scientific information include (1) time, (2) resource reliability, (3) trustworthiness/credibility of data, and (4) information overload.	#1
Hirschhorn et al. (2018) [64]	This paper describes reasons for the research to practice gap and offer suggestions to better bridge the chasm between researchers and implementers.	Research on quality improvement	Authors recommend a number of initial steps to better bridge the gap between researchers and implementers: (1) aligning project goals and joint planning; (2) choosing the right research design; (3) building implementer research capacity; (4) aligning incentives to drive collaboration; (5) simplifying documentation for dissemination of learning.	#5 #6
McKay et al. (2018) [65]	Given the infancy of de-implementation, this paper provides a conceptual narrative, definition, and criteria for determining if an intervention should be de-implemented	Human services research	Authors identify three criteria for identifying interventions appropriate for de-implementation: (a) interventions that are not effective or harmful, (b) interventions that are not the most effective or efficient to provide, and (c) interventions that are no longer necessary.	#1 #3
Kwon et al. (2018) [66]	This article seeks to provide understanding and examples of how to apply core principles of community-based participatory research in developing patient-centered outcomes research that can impact clinical and public health practice.	Community-based participatory research	Common themes of community-based participatory research and patient-centered outcomes research strategies are related to (1) fostering joint ownership in the identification of health priorities, development/evaluation of research strategies/design, and dissemination of findings; (2) recognition/appreciation for stakeholder priorities, research, and solutions; (3) building capacity of both stakeholders and researchers to engage in research collaboratively; and (4) recognizing that conducting the research is not the endpoint but continues with commitment to dissemination, spread, adoption, and sustainability.	#5 #8
Pettibone et al. (2018) [67]	The National Institute of Environmental Health Sciences (NIEHS) introduces a new translational research framework that builds upon previous biomedical models to create a more comprehensive and integrated environmental health paradigm.	Environmental health research	Adequate research is needed to test the effectiveness of interventions in real-world settings and adjusts the intervention accordingly to promote implementation. Authors also note the importance of (1) understanding health impact of an intervention in real-world settings and (2) community partner involvement and their input, knowledge, and skills for proper translation.	#1 #5 #8
Wathen and MacMillan (2018) [68]	This paper reviews key concepts in the knowledge translation area, with a particular focus on integrated knowledge translation, which focuses on researcher-knowledge user partnership in mental health and prevention of violence against women and children using examples from completed and ongoing work.	Research evidence	Importance of the 3Ts: (1) talk, (2) trust, and (3) time, in knowledge activities. That is, that developing and maintaining trusting partner relationships involves significant interaction, which takes time, and requires sustained effort and commitment by all involved. Uptake/use of new knowledge depends on how it resonated with stakeholders’ beliefs, values, experiences, and decision-making context. Active partner involvement throughout the research process improves the ability of the partnership to achieve its goals. Expanding the range of the potential knowledge users to health policy actors, health advocates, or the public.	#5 #11
Spiel et al. (2018) [69]	The authors propose an approach for the goal-oriented integration of intervention and implementation research.	Research knowledge	Systematic integration of intervention and implementation research and recommend a six-step procedure. Requires researchers to design/develop intervention programs using a field-oriented and participative approach. Policymaker perspectives must be included, and an analysis of which factors support evidence-based policy. Step 1: mission-driven problem recognition; step 2: ensuring availability of robust knowledge; step 3: identification of reasonable action starting points; step 4: establishment of a cooperation process with policymakers; step 5: coordinated development of intervention/implementation; step 6: transfer of program implementation.	#1 #5
Tait and Williamson (2019) [70]	The aim of this review is to determine the extent of the literature on training programs designed to improve researcher competency in knowledge translation and describe existing training methods that may be used by those hoping to build capacity for partnership research.	Knowledge translation training initiatives	Promising training themes that increase research translation include (1) increasing researchers’ knowledge and understanding of health policymaking processes, (2) improving understanding of knowledge translation research methods and knowledge translation theory, and (3) improving communication and relationship-building skills, and skills around the design and evaluation of knowledge translation plans.	#6
Close et al. (2019) [71]	The authors present an operational framework (the “Paper-2-Podium Matrix”) that provides a checklist of criteria for which to prompt the critical evaluation of performance nutrition-related research papers.	Nutrition research	Authors provided a time-efficient framework to aid practitioners in scientific appraisal of research to understand translation potential, by considering the (1) research context, (2) participant characteristics, (3) research design, (4) dietary and exercise controls, (5) validity and reliability of exercise performance tests, (6) data analytics, (7) feasibility of application, (8) risk/reward, and (9) timing of the intervention.	#1 #6
Mazzucca et al. (2019) [72]	The purpose of this study was to identify organizational supports for evidence-based decision-making within local health departments and determine psychometric properties of a measure of organizational supports.	Public health practice to prevent and control chronic disease	Factors influencing translation of evidence for decision-making: (1) awareness of culture supportive of evidence-based decision-making; (2) capacity and expectations for evidence-based decision-making; (3) resource availability; (4) evaluation capacity; (5) evidence-based decision-making climate cultivation; (6) partnerships to support evidence-based decision-making.	#5 #6 #11
Young et al. (2020) [73]	The purpose of this paper is to identify the characteristics that influence community-university partnerships and examine alignment with the Knowledge to Action Framework.	Community-university partnerships	Factors that influence community-university partnerships and translation: (1) adaptation (e.g., feasibility, participatory process, (2) transparency of motivating factors, (3) community engagement, (4) effectiveness (e.g., positive impacts shown in similar populations, (5) comparisons to existing programs), (6) evaluation (e.g., formative research, social assessments), (7) resources (e.g., human resources, back up plans for staff/funding changes, budgeting for engaging experts, existing human/financial resources), (8) stakeholders (e.g., considering end-users before translating, awareness of prior commitments, incorporation of local authorities/champions, leverage existing networks, buy-in from community advisory board), (9) respect for culture of setting (e.g., project is designed and suitable for community setting), and (10) trust/mutual respect (e.g., consideration of public image, active engagement for major decisions).	#1 #4 #5 #6 #9 #10 #11
Koh et al. (2020) [74]	This article seeks to frame and orient researchers from the behavioral sciences to the rapidly growing interdisciplinary field of dissemination and implementation science.	Research that is suitable for translation	Interventions should be empirically supported and effective in influencing health outcomes in the population of interest. They also be a good choice for the organization/setting of interest. Contextual factors to consider: (1) identifying/engaging stakeholders, (2) assessing acceptability, and (3) understanding organizational capacity, climate, and readiness to carry out the intervention.	#1 #3 #5
Barwick et al. (2020) [75]	This report summarizes advancements in knowledge translation practice generally, knowledge translation’s relationship with implementation science, and its practice in the specific area of disability research.	Disability research	For research translation, engaging stakeholders (in organizational components, values, and other practices), acknowledging external drivers (e.g., funding sources, academic promotion), and using theories/models/frameworks are important.	#5 #9
Morgan et al. (2020) [76]	Using the case study of a long-standing community-based participatory research project (“Investigaytors”), this article describes the development and implementation of a knowledge translation intervention aimed at facilitating access to HIV pre-exposure prophylaxis for gay, bisexual, and other sexual minority men in British Columbia, Canada, through a publicly funded program.	Community-based participatory research for a program that facilitates access to HIV pre-exposure prophylaxis	Important factors for knowledge translation: (1) inclusion of multiple perspectives from community, academic, and healthcare partners, and (2) perceived strength and credibility of the knowledge translation intervention opportunities for improving the community-based participatory research process, (3) understanding reciprocity that can include benefits such as training and professional development, and (4) introducing a novel approach to knowledge translation that is driven by community and integrates multiple perspectives.	#5

*CFIR* Consolidated Framework for Implementation Research, *RE-AIM* Reach, Effectiveness, Maintenance, Adoption, Implementation, and Maintenance framework, *PRECIS* Pragmatic-Explanatory Continuum Indicator Summary model

### Study characteristics — objective 1

The criteria for research translation used in past research outlined in Table 1 include important considerations that facilitate research translation, including contextual, organizational, or intervention characteristics. Several referenced one or more theoretical frameworks or models (*n*=22): ten used the Reach, Effectiveness, Maintenance, Adoption, Implementation, and Maintenance (RE-AIM) framework [20, 22, 37, 40, 41, 43, 52, 59, 60, 77], four used the Interactive Systems Framework [45–47, 50], two used Diffusion of Innovations theory [20, 52], two used CFIR [51, 58], one used the Predisposing, Reinforcing, and Enabling Constructs in Educational Diagnosis and Evaluation & Policy, Regulatory, and Organizational Constructs in Educational and Environmental Development (PRECEDE PROCEED) framework [41], one used the Pragmatic-Explanatory Continuum Indicator Summary (PRECIS) model [59], one used the National Institute of Environmental Health Research translational research framework [67], and one used the Evidence Integration Triangle model [59]. A total of 24 articles did not explicitly mention theoretical frameworks or models that were used to develop or apply research translation decision criteria.

### Result synthesis — objective 2

**Fig. 2:**
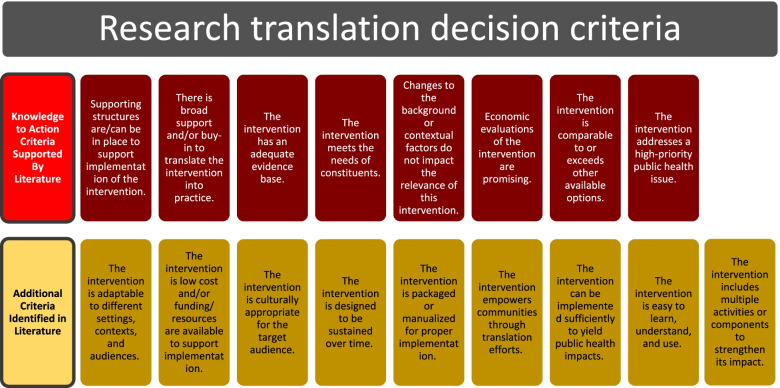
Final research translation decision criteria (rows ordered from most to least cited)

Table 2 and Fig. 2 display the 17 research translation decision criteria identified through our literature review and thematic analysis. Of the 8 themes from the K2A Framework that were used to inform the thematic analysis, all 8 were included in the final criteria list after research supported their importance for research translation decision-making (Table 2, criteria 1–8). Nine additional criteria were identified in the literature that were unrelated to the K2A Framework (Table 2, criteria 9–17). During the review phase, no additional criteria were added by the researchers or Community Advisory Board members. Wording changes were suggested by the Community Advisory Board members to improve the simplicity and coherency of select criteria. For example, Community Advisory Board members suggested that wording in one instance be changed from “does not disempower marginalized communities” to “empowers communities.” The following paragraphs describe each criterion, including citations for review articles that highlight their usefulness and application.

**Table 2:** Research translation decision criteria identified through the literature review

Number	Community-based translational criterion	Supporting references
1	The intervention has an adequate evidence base (efficacy, effectiveness, or implementation studies suggest that meaningful public health effects will result from translating the intervention into widespread use). ^a^K2A	(*n* = 20) [22, 37–41, 47, 49, 51, 56, 58–60, 63, 65, 67, 69, 71, 73, 74]
2	The intervention addresses a high-priority public health issue. ^a^K2A	(*n* = 2) [22, 59]
3	The intervention meets the needs of constituents. ^a^K2A	(*n* = 12) [21, 42, 44, 51–53, 56, 58, 60, 62, 65, 74]
4	The intervention is comparable to or exceeds other available interventions. ^a^K2A	(*n* = 5) [25, 41, 47, 51, 73]
5	There is broad support and/or buy-in to translate the intervention into practice. ^a^K2A	(*n* = 25) [21, 25, 33, 39, 40, 42, 44, 46, 47, 49, 51, 54, 57–59, 64, 66–69, 72–76]
6	There are supporting structures in place (or can be put in place) to support the implementation of the intervention (i.e., resources, training, technical assistance). ^a^K2A	(*n* = 26) [13, 14, 20–22, 25, 33, 39, 42, 44, 46, 47, 49–52, 54, 57, 58, 60, 62, 64, 70–73]
7	Economic evaluations of the intervention are promising (i.e., return on investment, cost benefit, cost-effectiveness). ^a^K2A	(*n* = 6) [22, 25, 47, 51, 55, 60]
8	Changes to the background or contextual factors (e.g., sociopolitical climate, time horizon) do not impact the relevance of this intervention or make it more relevant. ^a^K2A	(*n* = 8) [25, 40–42, 52, 60, 66, 67]
9	The intervention is very low cost and/or funding/resources are available to support its implementation.	(*n* = 14) [21, 22, 25, 33, 37, 42, 46, 51–53, 55, 58, 73, 75]
10	The intervention is adaptable to different settings, contexts, and audiences.	(*n* =1 5) [14, 21, 37, 41–44, 47, 51, 52, 55, 57, 59, 61, 73]
11	The intervention is culturally appropriate for the target audience.	(*n* = 8) [33, 45, 48, 51, 54, 68, 72, 73]
12	The intervention empowers communities through translation efforts.	(*n* = 3) [45, 48, 58]
13	The intervention includes multiple activities (components) to strengthen its impact.	(*n* = 1) [21]
14	The intervention is designed to be sustained over time.	(*n* = 6) [40, 42–44, 46, 52]
15	The intervention is easy to learn, understand, and use.	(*n* = 3) [21, 25, 42]
16	The intervention is packaged or “manualized” for proper implementation.	(*n* = 4) [42, 43, 52, 60]
17	There is evidence that the intervention can be implemented sufficiently to yield meaningful public health impacts.	(*n* = 3) [37, 52, 56]

The word “intervention” is used in a broad sense in the table and text to describe evidence-based programs, policies, interventions, guidelines, tool kits, strategies, and/or messages

^a^K2A labels criteria that were originally identified as part of the Knowledge to Action Framework [8]

As stated previously, the first 8 criteria in the list (Table 2, Fig. 2) were adapted from the K2A Framework. The *first criterion* is that the intervention has an adequate evidence base or that efficacy, effectiveness, or implementation studies demonstrate positive public health impacts of the intervention (e.g., research shows clinically significant benefits) [8]. Several articles (*n* = 20) identified in the literature search also noted this consideration for research translation [8, 22, 37–41, 47, 49, 51, 56, 58–60, 63, 65, 67, 69, 71, 73, 74], and specifically cited the importance of internal and external validity of evidence. The *second criterion* is that the intervention addresses a high-priority public health issue [8]. This means that the intervention addresses a chronic or acute disease affecting a significant portion of the population in the context where it is being implemented (*n* = 2 articles mentioned this) [8, 22, 59]. The *third criterion* is that the intervention meets the needs of constituents [8]. Specifically, it is important that the intervention fits with goals, norms, and practices of the population it is serving and is consistent with organizational and program needs (*n* = 12 articles mentioned this) [8, 21, 42, 44, 51–53, 56, 58, 60, 62, 65, 74]. Interventions must also maintain their same meaning after being adapted to fit the community needs [56]. The *fourth criterion* is that the intervention is comparable to or exceeds outcomes achieved from other available interventions [8]. The intervention should be better than available interventions or comparable but perhaps better suited for the context or population (i.e., there is a relative advantage) (*n* = 5 articles mentioned this) [8, 25, 41, 47, 51, 73]. The *fifth criterion* is that there is broad support and/or buy-in to translate the intervention into practice [8]. Research shows that the involvement of key stakeholders and implementation champions early on in the implementation process and proper organizational support are crucial for intervention success (*n* = 26 articles mentioned this) [8, 21, 25, 33, 39, 40, 42, 44, 46, 47, 49, 51, 54, 57–59, 64, 66–69, 72–76]. One article specifically noted the importance of having proper leadership support, with program goals and a vision that align [47]. The *sixth criterion* is that there are supporting structures in place (or can be put in place) to support the implementation of the intervention [8]. Resources, training programs, technical assistance, and time are necessary for proper translation, as well as the absence of competing demands, financial and organizational instability, and prevailing practices that work against the intervention goals (*n* = 26 articles mentioned this) [8, 14, 20–22, 25, 33, 39, 42, 44, 46, 47, 49–52, 54, 57, 58, 60, 62, 64, 70–73]. The *seventh criterion* deals with the economic evaluations of research translation, specifically that the intervention is cost-efficient and cost effective [8]. Interventions can be costly, and financial support can be hard to obtain or unreliable and can inhibit implementation efforts (*n* = 6 articles mentioned this) [8, 22, 25, 47, 51, 55, 60]. If funding and/or donations are available for translating the intervention, there should be some evidence that the investment will yield adequate public health impacts [8]. The *eighth criterion* adapted from K2A is that changes to the background or contextual factors do not adversely impact the relevance of the intervention [8]. The intervention must be amenable to changes in leadership, the sociopolitical climate, time-related barriers, or other contextual issues (*n* = 8 articles mentioned this) [8, 25, 40–42, 52, 60, 66, 67].

Additional criteria identified in the literature review are also displayed in Table 2 (and Fig. 2) and represent considerations that were not directly addressed in the K2A Framework. The *ninth criterion* is that the intervention must be very low cost or funding and resources must be available to sustain its implementation (*n* = 14 articles mentioned this) [21, 22, 25, 33, 37, 42, 46, 51–53, 55, 58, 73, 75]. In many contexts, funding may be scarce or unavailable but resource sharing options can promote translation and implementation [51]. The *tenth criterion* states that intervention must be adaptable to different settings, contexts, or audiences or at least the setting or context under study (*n* = 15 articles mentioned this) [14, 21, 37, 41–44, 47, 51, 52, 55, 57, 59, 61, 73]. This was a major consideration in included articles, since interventions were often translated in understudied locations/contexts. *Criterion eleven* underlines the importance that the intervention is culturally appropriate (*n* = 8 articles mentioned this) [33, 45, 48, 51, 54, 68, 72, 73], especially when health departments, academic units, or other external institutions with a history of unethical practices are involved [78–81]. As an example, researchers must continue to acknowledge past incidents, such as the Tuskegee Syphilis Study, that promote distrust for scientific institutions among populations of certain cultural, racial, and/or ethnic backgrounds [80]. Researchers cannot begin to understand and overcome low participation in public health research and programming without openly acknowledging the larger, historical context in which discrimination within the scientific community has led to mistreatment, denial of basic health care, and even death [45, 80, 81]. Adding to this, researchers must ensure interventions have culturally specific characteristics that may increase participation and positive reception [45]. *Criterion twelve* states that it is important that the intervention empowers communities through translation efforts (*n* = 3 articles mentioned this) [45, 48, 58]. Research translation should be community driven, meaning that local stakeholders and community members are ultimately autonomous over the intervention planning and participate in the translation process so that it properly addresses the community’s needs [45]. As well, *criterion thirteen* states that interventions that include multiple activities tend to be more effective because if one activity cannot be implemented, is not effective, or does not gain community interest, the intervention can still be carried out and have positive impacts (*n* = 1 article mentioned this) [21]. *Criteria fourteen, fifteen, and sixteen* relate to intervention sustainability: the intervention must be able to be sustained over time through strategic planning efforts (*n* = 6 articles mentioned this) [40, 42–44, 46, 52]; the intervention must be easy to learn, understand, and use for implementers and community members alike (*n* = 3 articles mentioned this) [21, 25, 42]; and the intervention should be packaged or manualized for easier implementation (*n* = 4 articles mentioned this) [42, 43, 52, 60]. Lastly, *criterion seventeen* states that there must be evidence that the intervention can be implemented sufficiently to yield meaningful public health impacts (*n* = 3 articles mentioned this) [37, 52, 56]. These can be shown through preliminary studies and can be assessed or considered before translation is initiated.

## Discussion

### Overall findings

This paper outlined the process used to identify and compile criteria that can be used by researchers and community partners to evaluate or compare interventions and their readiness for translation. As stated, the K2A Framework’s “decision to translate” planning questions, a literature review, a thematic analysis, and consideration of researcher and community partner input were used to develop these criteria. While there is growing interest in this research area, there is little current literature specifically on the *decision to translate* empirically informed interventions for public health and community-oriented researchers and practitioners, with only seven papers identified [38, 40, 42, 45, 56, 58, 75]. Additionally, there is a breadth of literature on factors that impact the research translation process more generally that were used to inform the resultant research translation decision criteria. Our literature review resulted in a final list of 17 criteria that may be useful for deciding whether an empirically informed intervention should be prioritized for translation in a community or organization. While each of these criteria is important, it is likely that an intervention will not meet all of those listed here. Nonetheless, these criteria can be used to assess readiness for translation by applying a holistic list of considerations to one intervention at a time, *or* they can be used to draw comparisons between well-researched interventions and assist with decision-making on which intervention should take precedence for translation efforts. Overall, the criteria identified through our review highlighted the importance of an intervention’s public health, cultural, and community relevance when considering its potential for research translation. Not only are intervention characteristics (e.g., evidence base, sustainability, cost) necessary to consider when contemplating introducing an intervention to the “real world,” it is also important to consider characteristics of the target setting and/or population (e.g., presence of supporting structures, support/buy-in).

### Comparison to past research

The criteria development outlined in this paper was informed by the K2A Framework, although there were important similarities and differences between that existing framework and the factors identified through our literature search. To start, economic evaluations were highlighted as particularly important in the K2A Framework [7, 8]. The importance of economic considerations was additionally supported by research identified from our literature review, specifically stating that the intervention must be low-cost and the community or organizational context must have funding and resources must be available to sustain its implementation [22, 25, 42, 51, 53, 55]. The K2A Framework also lists that interventions must be adaptable to contextual changes *over time* [7, 8]. Through our review, we discovered literature stating that the intervention must be adaptable to different settings, contexts, or audiences (and particularly those under study) [14, 21, 41–44, 47, 51, 61]. Next, K2A Framework research translation considerations state that the intervention must meet the needs of constituents [7, 8]. Research identified by our literature review expands on this, by highlighting the importance of culturally appropriate interventions that empower communities [45, 48, 58]. Here, the research pointed to the importance of autonomy for community members and stakeholders, which was not specifically outlined in the K2A Framework. As another example of our literature review identifying gaps, we built on the K2A highlighting that supporting structures (e.g., appropriate resources, training, technical assistance) must be in place [7, 8] by also including that interventions must be properly manualized to ensure quality assurance and control [42, 43]. Lastly, the K2A Framework states that interventions must address a high-priority public health issue [7, 8]. We learned throughout our literature search that an intervention must be able to be implemented sufficiently to yield meaningful public health impacts, which provides an additional consideration for understanding how to address the public health issue at hand [37, 52, 56]. In summary, there is important overlap and also clear distinctions between the criteria identified using the K2A Framework and the criteria developed through our literature review and consulting process.

Results from our review showed that several theoretical frameworks have been used in the past to make decisions around research translation, dissemination, implementation, and program planning [82, 83]. The frameworks identified in this study showed similarities and differences to the K2A Framework, which served as the foundation for our research translation decision criteria. For instance, the Consolidated Framework for Implementation Research (CFIR) promotes implementation theory development and research translation in community settings [51, 84, 85]. CFIR overlaps with the K2A Framework in important ways, and we integrated some CFIR components throughout our criteria development process. For instance, CFIR notes the importance of evaluating intervention evidence strength and quality, adaptability, complexity, packaging, and cost before deciding to translate or implement [51]. Moreover, CFIR states that inner and outer setting characteristics, such as the needs/resources of community members, external policies/incentives, and other structural or contextual factors, must be considered before deciding to translate an intervention to a specific community [51]. External policies, such as public funding opportunities for public health initiatives, and their impact on community buy-in and support are particularly important to consider for research translation [86]. Some citizens and communities even argue there is “overreach” by public health institutions when translating cost-intensive interventions, so it is crucial to properly justify the use of public funds and consider all points of view when evaluating the “contextual factors” outlined in our criteria list [86].

The RE-AIM framework was also identified in the literature on factors influencing research translation and is generally used as a method of systematically considering the strengths and weaknesses of interventions to guide program planning so that the public health impact is maximized [87]. Factors noted as important for research translation and implementation for RE-AIM include the cost of the intervention for community members, the potential for the intervention to yield positive outcomes (based on research), the presence of necessary resources and expertise within the community, the benefit of the intervention compared to existing programs, and the extent to which the intervention can be flexible and maintained over time [87]. Although this framework focuses more on contextual factors than intervention characteristics, these factors are important to consider before translating an intervention to a specific setting.

### Implications for research and practice

The literature review process and resultant research translation decision criteria outlined in this paper have important implications for public health research. First, public health researchers can study whether using our research translation decision criteria has an impact on intervention translation, implementation, dissemination, and maintenance in more diverse locations and applications. Researchers may even wish to develop ways to “grade” interventions on their ability to meet each criterion using a rating scale for comparison and to ensure assessments of whether an intervention meets related criteria are reliable. Second, researchers can identify whether our research translation decision criteria have a salient impact in specific communities or populations (i.e., those outside our local community where the criteria were developed), or for specific types of intervention (e.g., behavioral interventions, structural interventions). While creating a holistic list of research translation criteria is an important contribution to the literature, we recognize that some criteria may be less relevant depending on the context or intervention type, so researchers may wish to reduce the list to something less comprehensive or weight the importance of certain criteria more heavily for their application. Third, researchers should continue to identify intervention or community factors that impact research translation by reviewing relevant literature, collecting necessary data during intervention translation phases, and incorporating feedback from a more nationally representative group of experts and community leaders. Factors that impact research translation in community settings evolve, so they should be studied and monitored over time.

This study also has important implications for public health and community practice. To start, these research translation decision criteria can be applied by researchers and local stakeholders to initiate conversations on potential facilitators and barriers to translation into community settings. As an example, we identified an efficacious intervention (R01HL135220) conducted through our Prevention Research Center and asked the principal investigator to write a brief description of how the intervention met each of our 17 criteria to share with the Community Advisory Board. After considering the criteria and making comparisons with other grant options, the Community Advisory Board decided that the intervention (R01HL135220) was ready for translation and agreed to provide a letter of support for a grant application. Next, these criteria can be applied during earlier stages of the research process (before the research translation phase) so that researchers can design interventions that have adequate potential for translation. This practice is supported by the K2A Framework [7, 8] and a growing body of research aimed at “designing for dissemination,” [20, 52, 61] so that interventions are appropriate and sustainable in community settings. Additionally, considering these criteria may lead to additional research questions (e.g., what is the cost-effectiveness of this intervention?) that must be investigated before proceeding with research translation (a tactic also outlined within the K2A framework). Lastly, academic institutions, research centers, or federal agencies may wish to make policies that require public health researchers and practitioners to focus more on the decision to translate and consider developing criteria (using methods similar to ours) that capture factors that impact translation in their target communities. This provides a more consistent manner for evaluating whether an intervention is ready for translation in a community setting and may result in more reliable practices and consistent outcomes on an organizational level.

### Limitations and strengths

Limitations of this research should be noted. First, research translation decision criteria were based solely on peer-reviewed and published English-language articles, so information and related criteria from unpublished studies, non-English literature, or other communication forms (e.g., theses, government reports) are not represented. Second, this literature review only included reliability checking with a second reviewer during the full-text study selection phase. Although we acknowledge that this may have slightly decreased the number of relevant studies or themes identified [88], we made sure to employ pre-specified eligibility criteria, a systematic search strategy, and collaborative assessment and interpretation of findings. Third, due to limited research on the decision to translate research products, we incorporated research on the entire process for research translation, dissemination, and implementation. We believe that factors that influence more upstream, dissemination/implementation processes also play an important role in translation. Fourth, while this criteria development process involved over a dozen local community members and several researchers at our university’s Prevention Research Center, study findings and research translation decision criteria may lack generalizability. Future directions for these criteria may involve gaining more input from experts and community members in more diverse locations.

In addition to the noted limitations, this study has several strengths. To start, we made use of an existing framework (i.e., K2A Framework) to develop research translation decision criteria. Since this framework was developed by the CDC to describe and depict the high-level processes necessary to move from scientific knowledge and interventions into action [7], it served as a strong and empirically informed foundation for our criteria development. Another strength of this study is that it expands on the burgeoning field of research focusing on the *decision to translate* research interventions since there is a need for more research on this pivotal juncture between academic knowledge and community settings. Lastly, this study and its resultant research translation criteria were initiated to fill a specific need within a specific CDC-funded Prevention Research Center. Our criteria and related development methods can be used in similar Centers nationwide and in diverse locations to ensure researchers are bridging the gap between public health knowledge and action.

## Conclusion

This article provides a clear process for criteria development, by outlining our literature review, researcher input, and community engagement stages that were relevant to the research translation decision-making process. Our research translation decision criteria provide a holistic list for identifying important barriers and facilitators for research translation that can be considered before introducing an empirically supported intervention into community settings. Ideally, these criteria can serve as a novel tool for public health researchers and practitioners and provide more consistency in the process of research translation. Future directions for the development of these criteria may involve testing their use and seeking input from researchers and community leaders in more diverse locations to improve their generalizability.

## Data Availability

The data extracted from included studies are not publicly available due to possible copyright issues related to reproducing manuscript content but are available from the corresponding author on reasonable request.

## Abbreviations

WHO: World Health Organization
K2A: Knowledge to Action
CDC: Centers for Disease Control and Prevention
CFIR: Consolidated Framework for Implementation Research
RE-AIM: Reach, Effectiveness, Maintenance, Adoption, Implementation, and Maintenance
PRECEDE PROCEED: Predisposing, Reinforcing, and Enabling Constructs in Educational Diagnosis and Evaluation & Policy, Regulatory, and Organizational Constructs in Educational and Environmental Development
PRECIS: Pragmatic-Explanatory Continuum Indicator Summary

## References

[1] Forouzanfar MH, Afshin A, Alexander LT (2016). Global, regional, and national comparative risk assessment of 79 behavioural, environmental and occupational, and metabolic risks or clusters of risks, 1990–2015: a systematic analysis for the Global Burden of Disease Study 2015. Lancet.

[2] World Health Organization (2018). Noncommunicable diseases.

[3] Christensen K, Doblhammer G, Rau R, Vaupel JW (2009). Ageing populations: the challenges ahead. Lancet.

[4] Ince Yenilmez M (2015). Economic and social consequences of population aging the dilemmas and opportunities in the twenty-first century. Appl Res Qual Life.

[5] Population Reference Bureau. Fact sheet: aging in the United States. https://www.prb.org/aging-unitedstates-fact-sheet/. Accessed June 7, 2019.

[6] Rimer BK, Glanz K, Rasband G (2001). Searching for evidence about health education and health behavior interventions. Health Educ Behav.

[7] Wilson K, Brady T, Lesesne C, NCCDPHP Work Group on Translation. An organizing framework for translation in public health: the knowledge to action framework. Prev Chronic Dis. 2011;8(2) https://www.cdc.gov/pcd/issues/2011/mar/10_0012.htm. Accessed 21 Oct 2020. PMC3073439 21324260

[8] Centers for Disease Control and Prevention (2014). Applying the Knowledge to Action (K2A) framework: questions to guide planning.

[9] Zhao N, Koch-Weser S, Lischko A, Chung M (2020). Knowledge translation strategies designed for public health decision-making settings: a scoping review. Int J Public Health.

[10] Ashcraft LE, Quinn DA, Brownson RC (2020). Strategies for effective dissemination of research to United States policymakers: a systematic review. Implement Sci.

[11] Budd EL, deRuyter AJ, Wang Z (2018). A qualitative exploration of contextual factors that influence dissemination and implementation of evidence-based chronic disease prevention across four countries. BMC Health Serv Res.

[12] Rabin BA, Glasgow RE, Kerner JF, Klump MP, Brownson RC (2010). Dissemination and implementation research on community-based cancer prevention: a systematic review. Am J Prev Med.

[13] Davies P, Walker AE, Grimshaw JM (2010). A systematic review of the use of theory in the design of guideline dissemination and implementation strategies and interpretation of the results of rigorous evaluations. Implement Sci.

[14] Durlak JA, DuPre EP (2008). Implementation matters: a review of research on the influence of implementation on program outcomes and the factors affecting implementation. Am J Community Psychol.

[15] Hudson KG, Lawton R, Hugh-Jones S (2020). Factors affecting the implementation of a whole school mindfulness program: a qualitative study using the consolidated framework for implementation research. BMC Health Serv Res.

[16] Ecker AH, Abraham TH, Martin LA, Marchant-Miros K, Cucciare MA. Factors affecting adoption of Coordinated Anxiety Learning and Management (CALM) in Veterans’ Affairs community-based outpatient clinics. J Rural Health. 2020:jrh.12528. 10.1111/jrh.12528. 10.1111/jrh.12528 PMC8293288 33078451

[17] Seward K, Finch M, Yoong SL (2017). Factors that influence the implementation of dietary guidelines regarding food provision in centre based childcare services: a systematic review. Prev Med.

[18] Naylor PJ, Nettlefold L, Race D (2015). Implementation of school based physical activity interventions: a systematic review. Prev Med.

[19] Hage E, Roo JP, van Offenbeek MA, Boonstra A (2013). Implementation factors and their effect on e-Health service adoption in rural communities: a systematic literature review. BMC Health Serv Res.

[20] Brownson RC, Jacobs JA, Tabak RG, Hoehner CM, Stamatakis KA (2013). Designing for dissemination among public health researchers: findings from a national survey in the United States. Am J Public Health.

[21] Baker EA, Brennan Ramirez LK, Claus JM, Land G (2008). Translating and disseminating research- and practice-based criteria to support evidence-based intervention planning. J Public Health Manag Pract.

[22] Glasgow RE, Klesges LM, Dzewaltowski DA, Bull SS, Estabrooks P (2004). The future of health behavior change research: what is needed to improve translation of research into health promotion practice?. Ann Behav Med.

[23] Albrecht L, Archibald M, Arseneau D, Scott SD (2013). Development of a checklist to assess the quality of reporting of knowledge translation interventions using the Workgroup for Intervention Development and Evaluation Research (WIDER) recommendations. Implement Sci.

[24] Flay BR, Biglan A, Boruch RF (2005). Standards of evidence: criteria for efficacy, effectiveness and dissemination. Prev Sci.

[25] Ross J, Stevenson F, Lau R, Murray E (2016). Factors that influence the implementation of e-health: a systematic review of systematic reviews (an update). Implement Sci.

[26] King E, Boyatt R (2015). Exploring factors that influence adoption of e-learning within higher education: factors that influence adoption of e-learning. Br J Educ Technol.

[27] Skinner JS, Williams NA, Richmond A (2018). Community experiences and perceptions of clinical and translational research and researchers. Prog Community Health Partnersh Res Educ Action.

[28] Gusenbauer M (2019). Google Scholar to overshadow them all? Comparing the sizes of 12 academic search engines and bibliographic databases. Scientometrics.

[29] PubMed.gov. National Library of Medicine. https://pubmed.ncbi.nlm.nih.gov/. Accessed 22 Sept 2021.

[30] Bauer MS, Kirchner J (2020). Implementation science: what is it and why should I care?. Psychiatry Res.

[31] Glasgow RE, Vogt TM, Boles SM (1999). Evaluating the public health impact of health promotion interventions: the RE-AIM framework. Am J Public Health.

[32] The Community Guide (2015). Glossary.

[33] Mendel P, Meredith LS, Schoenbaum M, Sherbourne CD, Wells KB (2008). Interventions in organizational and community context: a framework for building evidence on dissemination and implementation in health services research. Adm Policy Ment Health Ment Health Serv Res.

[34] Lencucha R, Kothari A, Hamel N (2010). Extending collaborations for knowledge translation: lessons from the community-based participatory research literature. Evid Policy J Res Debate Pract.

[35] Fereday J, Muir-Cochrane E (2006). Demonstrating rigor using thematic analysis: a hybrid approach of inductive and deductive coding and theme development. Int J Qual Methods.

[36] Hamel C, Michaud A, Thuku M (2021). Defining Rapid Reviews: a systematic scoping review and thematic analysis of definitions and defining characteristics of rapid reviews. J Clin Epidemiol.

[37] Glasgow RE (2003). Translating research to practice: lessons learned, areas for improvement, and future directions. Diabetes Care.

[38] Dzewaltowski DA, Estabrooks PA, Glasgow RE (2004). The future of physical activity behavior change research: what is needed to improve translation of research into health promotion practice?. Exerc Sport Sci Rev.

[39] Glasgow RE, Marcus AC, Bull SS, Wilson KM (2004). Disseminating effective cancer screening interventions. Cancer.

[40] Klesges LM, Estabrooks PA, Dzewaltowski DA, Bull SS, Glasgow RE (2005). Beginning with the application in mind: designing and planning health behavior change interventions to enhance dissemination. Ann Behav Med.

[41] Green LW, Glasgow RE (2006). Evaluating the relevance, generalization, and applicability of research: issues in external validation and translation methodology. Eval Health Prof.

[42] Glasgow RE, Emmons KM (2007). How can we increase translation of research into practice? Types of evidence needed. Annu Rev Public Health.

[43] Prohaska TR, Peters KE (2007). Physical activity and cognitive functioning: translating research to practice with a public health approach. Alzheimers Dement.

[44] Flaspohler P, Duffy J, Wandersman A, Stillman L, Maras MA (2008). Unpacking prevention capacity: an intersection of research-to-practice models and community-centered models. Am J Community Psychol.

[45] Guerra NG, Knox L (2008). How culture impacts the dissemination and implementation of innovation: a case study of the families and schools together program (FAST) for preventing violence with immigrant Latino youth. Am J Community Psychol.

[46] Livet M, Courser M, Wandersman A (2008). The prevention delivery system: organizational context and use of comprehensive programming frameworks. Am J Community Psychol.

[47] Wandersman A, Duffy J, Flaspohler P (2008). Bridging the gap between prevention research and practice: the interactive systems framework for dissemination and implementation. Am J Community Psychol.

[48] Scharff DP, Mathews K (2008). Working with communities to translate research into practice. J Public Health Manag Pract.

[49] Arrington B, Kimmey J, Brewster M (2008). Building a local agenda for dissemination of research into practice. J Public Health Manag Pract.

[50] Saul J, Duffy J, Noonan R (2008). Bridging science and practice in violence prevention: addressing ten key challenges. Am J Community Psychol.

[51] Damschroder LJ, Aron DC, Keith RE, Kirsh SR, Alexander JA, Lowery JC (2009). Fostering implementation of health services research findings into practice: a consolidated framework for advancing implementation science. Implement Sci.

[52] Prochaska JJ, Fromont SC, Hudmon KS, Cataldo JK (2009). Designing for dissemination: development of an evidence-based tobacco treatment curriculum for psychiatry training programs. J Am Psychiatr Nurses Assoc.

[53] Green LW, Ottoson JM, García C, Hiatt RA (2009). Diffusion theory and knowledge dissemination, utilization, and integration in public health. Annu Rev Public Health.

[54] Van Olphen J, Green L, Barlow J, Koblick K, Hiatt R (2009). Evaluation of a partnership approach to translating research on breast cancer and the environment. Prog Community Health Partnersh Res Educ Action.

[55] Ritzwoller DP, Sukhanova A, Gaglio B, Glasgow RE (2009). Costing behavioral interventions: a practical guide to enhance translation. Ann Behav Med.

[56] Sousa VD, Rojjanasrirat W (2011). Translation, adaptation and validation of instruments or scales for use in cross-cultural health care research: a clear and user-friendly guideline: validation of instruments or scales. J Eval Clin Pract.

[57] Meyers DC, Durlak JA, Wandersman A (2012). The quality implementation framework: a synthesis of critical steps in the implementation process. Am J Community Psychol.

[58] Cilenti D, Brownson RC, Umble K, Erwin PC, Summers R (2012). Information-seeking behaviors and other factors contributing to successful implementation of evidence-based practices in local health departments. J Public Health Manag Pract.

[59] Glasgow RE (2013). What does it mean to be pragmatic? Pragmatic methods, measures, and models to facilitate research translation. Health Educ Behav.

[60] Phillips SM, Alfano CM, Perna FM, Glasgow RE (2014). Accelerating translation of physical activity and cancer survivorship research into practice: recommendations for a more integrated and collaborative approach. Cancer Epidemiol Biomark Prev.

[61] Cohen EL, Head KJ, McGladrey MJ (2015). Designing for dissemination: lessons in message design from “1-2-3 Pap”. Health Commun.

[62] Neta G, Glasgow RE, Carpenter CR (2015). A framework for enhancing the value of research for dissemination and implementation. Am J Public Health.

[63] Brownson RC, Eyler AA, Harris JK, Moore JB, Tabak RG (2018). Getting the word out: new approaches for disseminating public health science. J Public Health Manag Pract.

[64] Hirschhorn LR, Ramaswamy R, Devnani M, Wandersman A, Simpson LA, Garcia-Elorrio E (2018). Research versus practice in quality improvement? Understanding how we can bridge the gap. Int J Qual Health Care.

[65] McKay VR, Morshed AB, Brownson RC, Proctor EK, Prusaczyk B (2018). Letting go: conceptualizing intervention de-implementation in public health and social service settings. Am J Community Psychol.

[66] Kwon SC, Tandon SD, Islam N, Riley L, Trinh-Shevrin C (2018). Applying a community-based participatory research framework to patient and family engagement in the development of patient-centered outcomes research and practice. Transl Behav Med.

[67] Pettibone KG, Balshaw DM, Dilworth C (2018). Expanding the concept of translational research: making a place for environmental health sciences. Environ Health Perspect.

[68] Wathen CN, MacMillan HL (2018). The role of integrated knowledge translation in intervention research. Prev Sci.

[69] Spiel C, Schober B, Strohmeier D (2018). Implementing intervention research into public policy—the “I3-Approach”. Prev Sci.

[70] Tait H, Williamson A (2019). A literature review of knowledge translation and partnership research training programs for health researchers. Health Res Policy Syst.

[71] Close GL, Kasper AM, Morton JP (2019). From paper to podium: quantifying the translational potential of performance nutrition research. Sports Med.

[72] Mazzucca S, Parks RG, Tabak RG (2019). Assessing organizational supports for evidence-based decision making in local public health departments in the United States: Development and psychometric properties of a new measure. J Public Health Manag Pract.

[73] Young BR, Leeks KD, Bish CL (2020). Community-university partnership characteristics for translation: evidence from CDC’s prevention research centers. Front Public Health.

[74] Koh S, Lee M, Brotzman LE, Shelton RC. An orientation for new researchers to key domains, processes, and resources in implementation science. Transl Behav Med. 2018. 10.1093/tbm/iby095. 10.1093/tbm/iby095 30445445

[75] Barwick M, Dubrowski R, Petricca K (2020). Knowledge translation: the rise of implementation.

[76] Morgan J, Schwartz C, Ferlatte O, et al. Community-based participatory approaches to knowledge translation: HIV prevention case study of the investigaytors program. Arch Sex Behav. 2020. 10.1007/s10508-020-01789-6. 10.1007/s10508-020-01789-6 32737658

[77] Dzewaltowski DA, Glasgow RE, Klesges LM, Estabrooks PA, Brock E (2004). RE-AIM: evidence-based standards and a web resource to improve translation of research into practice. Ann Behav Med.

[78] Pacheco CM, Daley SM, Brown T, Filippi M, Greiner KA, Daley CM (2013). Moving forward: breaking the cycle of mistrust between American Indians and researchers. Am J Public Health.

[79] Geronimus AT, Thompson JP. To denigrate, ignore, or disrupt: racial inequality in health and the impact of a policy-induced breakdown of African American communities. Bois Rev Soc Sci Res Race. 2004;1(02). 10.1017/S1742058X04042031.

[80] Gamble VN (1997). Under the shadow of Tuskegee: African Americans and health care. Am J Public Health.

[81] Pirie A, Gute DM (2013). Crossing the chasm of mistrust: collaborating with immigrant populations through community organizations and academic partners. Am J Public Health.

[82] Fernandez ME, Ruiter RAC, Markham CM, Kok G (2019). Intervention mapping: theory- and evidence-based health promotion program planning: perspective and examples. Front Public Health.

[83] Colquhoun HL, Letts LJ, Law MC, MacDermid JC, Missiuna CA (2010). A scoping review of the use of theory in studies of knowledge translation. Can J Occup Ther.

[84] Breimaier HE, Heckemann B, Halfens RJG, Lohrmann C (2015). The Consolidated Framework for Implementation Research (CFIR): a useful theoretical framework for guiding and evaluating a guideline implementation process in a hospital-based nursing practice. BMC Nurs.

[85] The Consolidated Framework for Implementation Research – technical assistance for users of the CFIR framework. CFIR. https://cfirguide.org/. Accessed 21 Feb 2022.

[86] Hunter EL (2016). Politics and public health—engaging the third rail. J Public Health Manag Pract.

[87] Glasgow RE, McKay HG, Piette JD, Reynolds KD (2001). The RE-AIM framework for evaluating interventions: what can it tell us about approaches to chronic illness management?. Patient Educ Couns.

[88] Stoll CRT, Izadi S, Fowler S, Green P, Suls J, Colditz GA (2019). The value of a second reviewer for study selection in systematic reviews. Res Synth Methods.

